# Time-Restricted Feeding Extends Healthspan in Female and Lifespan in Male C57BL/6J Mice

**DOI:** 10.1101/2025.10.22.683527

**Published:** 2025-10-23

**Authors:** Samantha E. Iiams, Nathan J. Skinner, Victoria A. Acosta-Rodríguez, Mary Wight-Carter, Carla B. Green, Joseph S. Takahashi

**Affiliations:** 1 Department of Neuroscience, Peter O’Donnell Jr. Brain Institute, University of Texas Southwestern Medical Center, Dallas, TX, USA; 2 Howard Hughes Medical Institute, University of Texas Southwestern Medical Center, Dallas, TX, USA; 3 Animal Resource Center, University of Texas Southwestern Medical Center, Dallas, TX, USA

## Abstract

Time-restricted feeding (TRF) aligned with an organism’s circadian rhythm has been shown to improve health, but its long-term effects on healthspan and lifespan in mammals, especially under normal dietary conditions, remain unclear. In this study, we tested the impact of 12-hour (h) and 8h nightly TRF windows on aging in male and female mice fed regular chow. TRF improved several health measures, including the regularity of behavioral patterns, body weight and composition, frailty, and disease onset, with stronger effects observed in the 8h-TRF group, which self-imposed a caloric restriction. When integrated into a composite Healthspan Index, these improvements revealed that TRF prolonged healthspan in females more effectively than in males. Only 8h-TRF extended lifespan in males by 12%, suggesting that TRF exerts sex-specific effects on aging.

While human life expectancy has nearly doubled since the early 20th century (1), healthspan remains limited, with many spending over 10% of their lives in poor health due to age-related functional declines within tissue and the accompanying development of disease (2). Caloric restriction (CR), a potent aging intervention involving a 30–50% reduction in food intake without malnutrition, has been extensively studied for its ability to delay the onset of age-associated diseases, including cancer, cardiovascular disorders, and neurodegeneration, while extending lifespan across multiple model organisms (3–5). Notably, incorporating daily feeding/fasting cycles with CR enhances these geroprotective effects (6–9), and aligning feeding with the active phase of an organism’s circadian rhythm further extends lifespan (10). This synergy likely arises from both synchronizing food intake with internal metabolic processes and from the ability of feed-fasting rhythms to enhance daily oscillations in the expression of genes regulated by circadian clocks in the liver and other peripheral tissues (11–17).

With age, circadian rhythms in behavior and physiology weaken as the amplitude of clock-controlled gene expression damps or shifts in phase (18). However, interventions that rescue circadian rhythms have been shown to improve health and extend longevity (19). Thus, optimization of meal timing presents a promising strategy to counteract aging-related rhythm disruptions and promote healthy longevity.

Given that CR can be challenging to maintain in people (20), meal timing alone via time-restricted feeding (TRF), or time-restricted eating (TRE) in humans, has emerged as an alternative strategy to delay aging (21, 22). TRF, which involves daily windows of feeding and fasting without imposing CR, confers similar benefits as timed CR, including improved rhythmic gene expression, cognitive function, and cardiometabolic health, even in models of obesity fed a high-fat, high-sucrose diet (23–29). Clinical trials testing TRE have noted high adherence rates and improvements in cardiometabolic health, altogether demonstrating its potential as a feasible and potentially effective intervention to enhance human healthspan (30–32). However, few studies have examined the benefits of TRF/TRE under non-obese conditions (33–35), and even fewer have compared impacts between the sexes (36). The long-term impact of TRF on healthspan and lifespan in mammals also remains unclear (37, 38). In this study, we sought to address these key knowledge gaps and determine whether TRF could serve as an effective aging intervention for healthy males and females.

## Results:

### Early onset circadian aligned TRF has sex-specific impacts on longitudinal feeding and wheel-running activity levels

**Figure 1:**
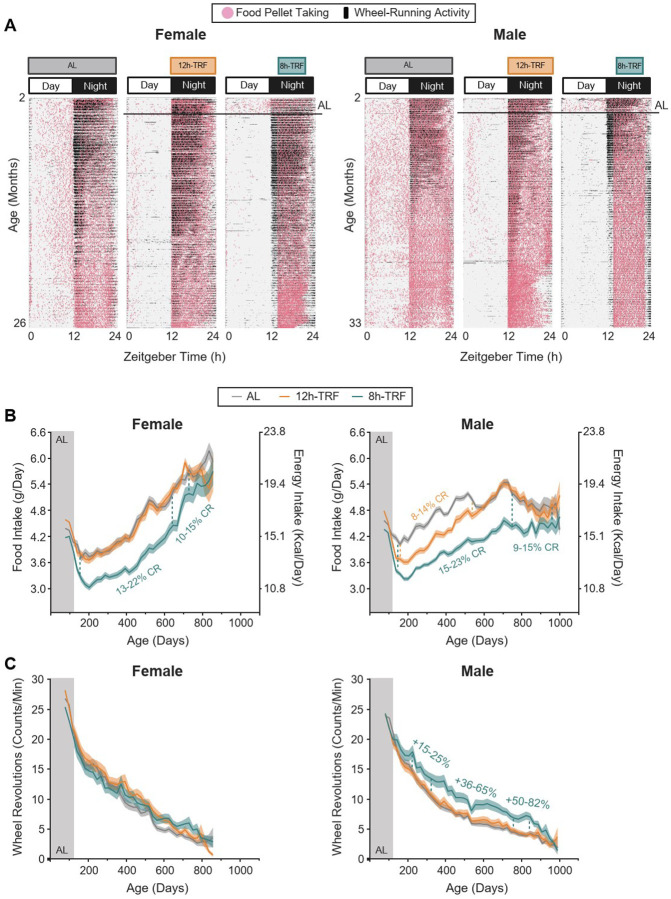
Circadian aligned TRF reduces feeding and improves wheel-running activity in a feeding window- and sex-dependent manner. (**A**) In females 2–26 months of age and males 2–33 months of age, representative actograms of individual mice showing wheel-running (black mark) and food pellet taking (pink dot) activity over the course of the 24-hour light:dark cycle. Black line shows ends of *ad libitum* (AL) baseline feeding for the TRF groups at 4 months of age. (**B**) Longitudinal profiles of average daily food intake and energy intake with age. (**C**) Longitudinal profiles of average daily wheel revolutions with age. B and C: AL baseline feeding (Gray area). TRF feeding conditions (White area). Each point represents mean of 21 days ± SEM (shaded regions). Significant differences TRF vs AL shown as % difference (P < 0.05) as determined by two-way ANOVA, Tukey’s post-hoc. Females N=71–103 per group. Males N=71–97 per group.

To determine how time-restricted feeding (TRF) impacts healthspan and lifespan in mammals, we individually housed 264 female and 264 male C57BL/6J mice in wheel cages with automated feeders at 2 months of age. To measure impacts under normal nutritional conditions, mice were fed purified precision pellets (Bioserv F0075). For the first 8-weeks, all mice were fed *ad libitum* (free-feeding; AL) with access to a maximum of 22 pellets or 6.6g (23.8 Kcal) per day. At 4 months of age, N=78/sex were switched to a 12-hour (h) TRF window (Zeitgeber time (ZT) 12–24) and another N=78/sex to an 8h-TRF window (ZT14–22) both circadian aligned to the night, the active phase of nocturnal rodents. The remaining N=108/sex were maintained AL to serve as the control. Across all feeding groups mice had access to the same amount of food, so as not to impose caloric restriction (CR), and were maintained on these regimens for life (Fig. 1A and Fig. S1) (39).

Measurements of daily food intake throughout each mouse’s lifespan showed that feeding increased over time in both sexes, then declined near the end of life in males, similar to a previous study (Fig. 1, A and B, and Fig. S2–3) (39). In line with other longitudinal recordings (40), females in our study consumed more food relative to their body weight at older ages than male. We also observed sex-specific responses to TRF where 12h-TRF females consumed the same amount as AL, while 12h-TRF males significantly reduced intake compared to AL by 8–14% from 140–539 days (~5–18 months) (two-way ANOVA: Tukey’s post-hoc, P ≤ 0.05). In contrast, 8h-TRF self-imposed CR for the majority of life in both sexes, with intake in females reduced by 10–22% between 140–728 days (~5–24 months) and males reduced by 9–23% between 140–980 days (~5–33 months) relative to sex- and age-matched AL controls. This 8h-TRF schedule, which narrows the daily eating window without explicitly limiting calorie intake, as seen in clinical trials (41), presents an alternative strategy for achieving long-term CR in both sexes.

Analysis of daily wheel-running recordings for each mouse revealed that individuals in all feeding groups, of both sexes, consistently displayed nocturnal activity, which subsequently declined with age, as anticipated (Fig. 1, A and C and Figs. S4–5) (42, 43). Only 8h-TRF males had increased levels of wheel activity from 224–833 days of age (~7–28 months) with the enhanced activity ranging +36–82% from 329–833 days (~11–28 months) relative to AL (two-way ANOVA: Tukey’s post-hoc, P ≤ 0.05). This male-specific difference further underscores the sex-specific effects of TRF on behavior. Additionally, in line with previous work (10, 44), the sustained increases in activity under 8h-TRF suggest a healthspan-enhancing effect in males.

### TRF improves the amplitude of daily rhythmic behaviors

**Figure 2:**
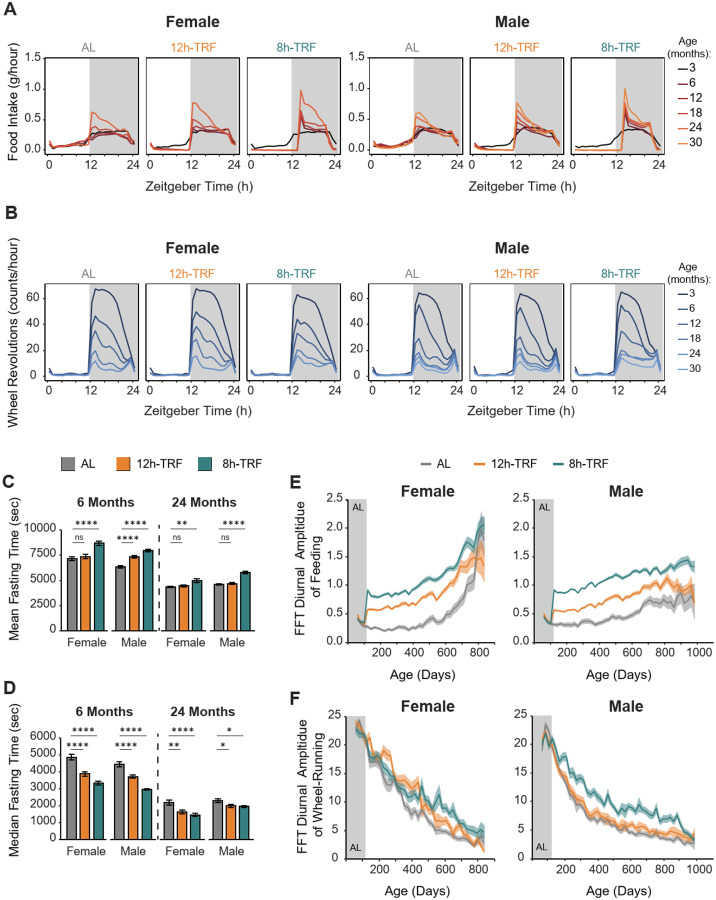
TRF improves the amplitude of daily feeding and wheel-running rhythms. (**A**) Twenty-four-hour profiles of feeding and (**B**) wheel-running starting at 3 and then every 6 months of age. 12h light (white area), 12h dark (gray area). (**C**) Mean and (**D**) median fasting times (sec) throughout the feeding/fasting cycle at 6 and 24 months of age. Data represented as mean ± SEM. Significant differences TRF vs AL shown with asterisks (P < 0.05) as determined by two-way ANOVA and Holm’s post-hoc. (**E**) Longitudinal FFT plots of the diurnal amplitude of feeding and (**F**) wheel-running with age. AL baseline feeding (Gray area). TRF feeding conditions (White area). Each point represents mean of 15 days ± SEM (shaded regions). For all panels: Females N=71–103 per group. Males N=71–97 per group.

We next examined age-related changes in the diurnal profiles of feeding and wheel-running, two well-established rhythmic behaviors, as high-amplitude behavioral rhythms are closely associated with improved health outcomes (Fig. 2, A and B) (18, 19, 45, 46). Across all groups, food intake became consolidated to the night, from 6 to 24 months of age. Both mean and median fasting durations, which account for daytime fasting and the short intervals between pellets during the feeding phase, declined with the most significant consolidation observed in the 8h-TRF group (Fig. 2, A, C, and D). A Fast Fourier Transform (FFT) analysis of diurnal amplitude, reflecting the strength of daily rhythms, unsurprisingly confirmed that TRF increased feeding amplitude and lead to a preservation of enhanced rhythmicity with age, particularly in the 8h-TRF group. (Fig. 2, A and E). While the diurnal amplitude of wheel-running declined with age across all groups, 8h-TRF males maintained stronger rhythms throughout life compared to controls (Fig. 2, B and F). Females in both TRF groups also showed modest increases in wheel-running amplitude, with 8h-TRF sustaining enhanced rhythms relative to controls from 350 days of age (~12 months) through the end of life. Together, these findings highlight that distinct behavioral rhythms, such as feeding and activity, are differentially affected by aging.

### TRF reduces age-related gains in body weight and fat

**Figure 3:**
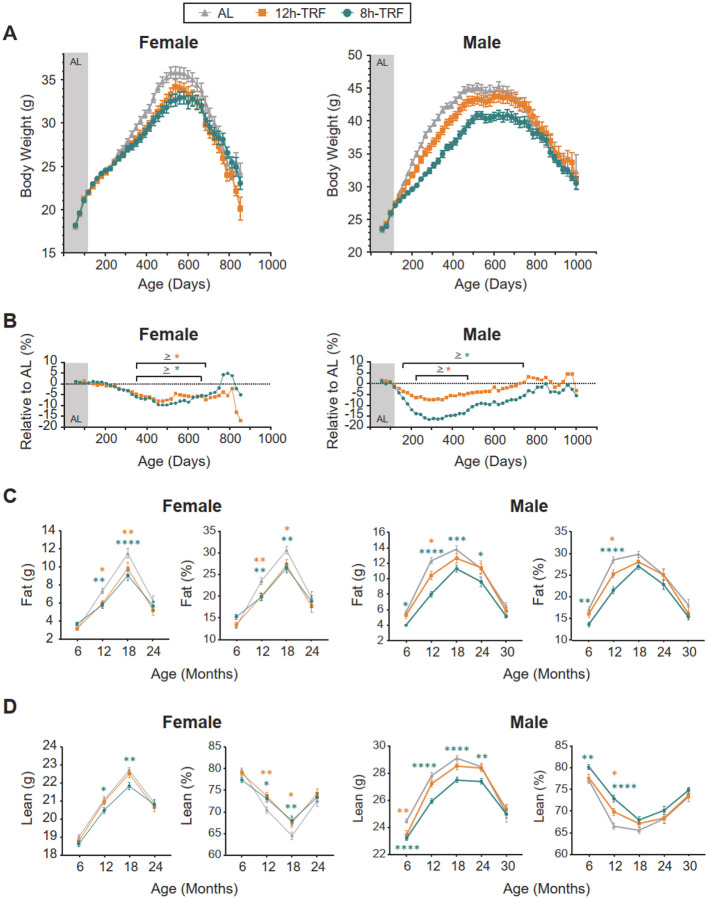
A 12h TRF window is sufficient to reduce body weight and fat mass in both sexes. (**A**) Longitudinal profiles of body weight and (**B**) percent differences in body weight relative to AL controls with age. For panels A and B: AL baseline feeding (Gray area). TRF feeding conditions (White area). Group means every 21 days (at cage change) ± SEM (bars). Females N=76–103 per group. Males N=76–102 per group. (**C**) Total fat mass and percentage of body weight with age. (**D**) Total lean mass and percentage of body weight with age. For panels C and D: Group means every 6 months ± SEM (bars). Females 6–24 months: AL, N=43–101. 12h-TRF, N=32–71. 8h-TRF, N=38–72. Males 6–30 months: AL, N=23–102. 12h-TRF, N=19–77. 8h-TRF, N=36–75. For all panels: Significant differences TRF vs AL shown with colored asterisks (P < 0.05; Orange=12h-TRF; Teal=8h-TRF) as determined by two-way ANOVA, Tukey’s post-hoc.

Body weights were recorded every 21 days (Fig. 3, A and B, and Fig. S6), and body composition was analyzed every 6 months using nuclear magnetic resonance imaging (EchoMRI, Houston, TX) (Fig. 3, C and D). In females, 12h-TRF without CR still weakened age-related gains in body weight by 5–8% from 350–686 days of age (~12–23 months) (two-way ANOVA: Tukey’s post-hoc, P ≤ 0.05). This feeding window also improved fat vs lean body composition, reducing fat mass while increasing lean mass by 3–4% at 12 and 18 months of age. Unexpectedly, 8h-TRF females with self-imposed CR did not exhibit any further improvements beyond 12h-TRF, in weight or fat/lean composition. In males, 12h-TRF reduced body weight gain by 5–7% from 224–476 days (~7–16 months) and improved fat vs lean composition by 3% at 12 months. 8h-TRF in males enhanced these benefits, lowering body weight gain by 5–16% from 161–749 days (~5–25 months) and improving fat vs lean composition by 3–7% from 6–12 months. Overall, this shows that 12h-TRF is sufficient to attenuate body weight gain and improve body composition in both sexes, with longer-term benefits in 12h-TRF females, but that 8h-TRF with long-term CR provides enhanced benefits for males.

### TRF slows age-related increases in frailty index values

**Figure 4:**
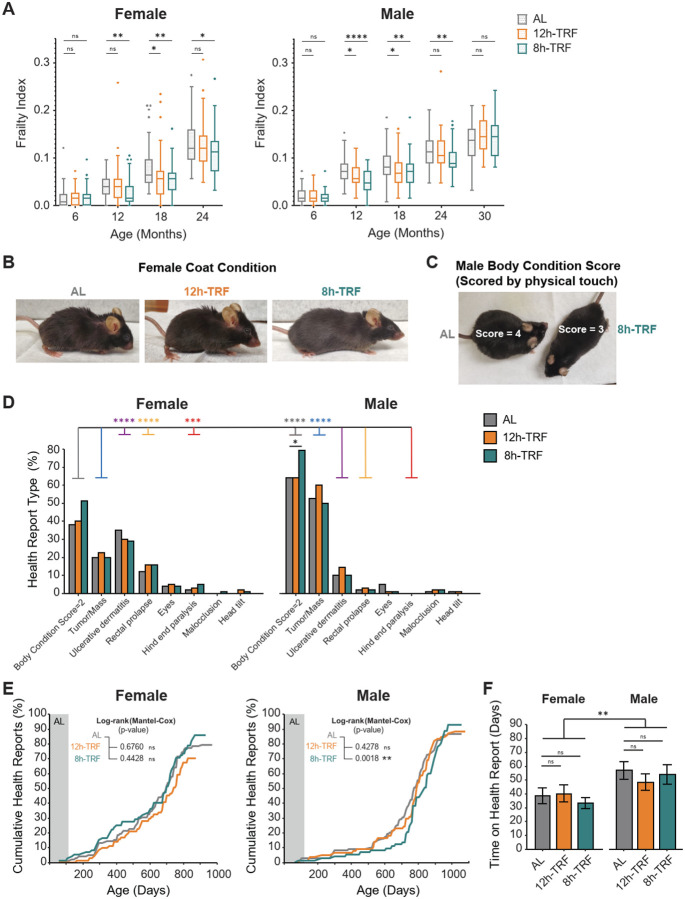
TRF reduces frailty index in both sexes but only delays onset of health reports in males. (**A**) Boxplots of frailty index scores every 6 months. Females: AL, N=44–79. 12h-TRF, N=34–74. 8h-TRF, N=40–74. Males: AL, N=23–79. 12h-TRF, N=22–78. 8h-TRF, N=39–76. Significant differences TRF vs AL shown with asterisks (P < 0.05) as determined by two-way ANOVA, Tukey’s post-hoc. (**B**) One example parameter contributing to reduced frailty index in 8h-TRF females: coat condition, P < 0.05 at 24 months compared to AL. (Full list see Fig. S7). (**C**) One example parameter contributing to reduced frailty index in 8h-TRF males: body condition score, P < 0.05 12–18 months compared to AL. (Full list see Fig. S8). (**D**) Percentage of mice in each feeding group and sex reported to veterinary staff for Body Condition Score=2, Tumor/Mass, Ulcerative dermatitis, Rectal prolapses, Eye ulcerations/swelling, Hind end paralysis, Malocclusions, and Head tilts. Significant differences TRF vs AL shown with black asterisk (P < 0.05; Body Condition Score=2) and females vs males with colored asterisks (P < 0.05; Gray= Body Condition Score=2; Blue=Tumor/Mass; Purple=Ulcerative dermatitis; Yellow=Rectal prolapse; and Red=Hind end paralysis) as determined by contingency tables and Fisher’s exact tests. (**E**) Cumulative curve showing the percentage of veterinary health reports filed in each group with age. Log-Rank Mantel-Cox Test for significant difference in overall curve TRF vs AL. (**F**) Time spent on health report until death. Data shown as mean ± SEM and significant differences with asterisks (P<0.05) as determined by two-way ANOVA, Holm’s post-hoc.

Frailty, a widely used measure of healthspan in aging mice, integrates 31 physiological and behavioral parameters into a composite health index (47). Frailty assessments were conducted every 6 months and showed that TRF significantly reduced frailty index scores, with varying impacts between the two feeding windows (Fig. 4, A–C, and Figs. S7–8). In females, 12h-TRF significantly reduced frailty index scores at 18 months, with hearing loss and body condition assessments defining the key differences between the two groups. 8h-TRF further extended reductions in frailty from 12 to 24 months, with improvements observed in grimace, piloerection, forelimb grip strength, coat condition, ulcerative dermatitis, and body condition scores. Males showed similar trends: 12h-TRF reduced frailty indices between 12 and 18 months, primarily improving grimace and body condition, while 8h-TRF sustained these benefits through 24 months and improved additional parameters, including grimace, hearing, piloerection, menace reflex, fur color loss, coat condition, and body condition. Although both TRF regimens reduced frailty, supporting the intervention’s effectiveness in extending healthspan, the responses were dose-dependent to the degree of TRF, with 8h-TRF providing the most prolonged benefits and improving the greatest number of health measures.

### The age-related onset of health reports reveals sex-specific differences and a delayed onset in 8h-TRF males

As mice developed health issues they were reported to veterinary staff for more frequent monitoring. These reports revealed that TRF did not reduce the relative frequency of commonly reported ailments compared to AL (Fig. 4D). In fact, 8h-TRF males has significantly more reports of body condition scores declining to 2 (Scored 1–5 with: 1 being emaciated, 3 ideal condition, and 5 obese) (Fisher’s exact test 8h-TRF vs AL: Body Condition Score=2, P < 0.05). However, we observed a clear sex-dependent pattern in the types and frequencies of reported conditions. Females, regardless of feeding group, were significantly more likely to be reported for ulcerative dermatitis (Fisher’s exact test females vs males: P < 0.0001), rectal prolapse (P < 0.0001), and hind limb paralysis (often associated with spinal tumors, P < 0.001). In contrast, males were more frequently reported for external tumors and abdominal masses (Fisher’s exact test females vs males: P < 0.0001), as well as Body Condition Score=2 (P < 0.0001). The onset of age-related health issues also differed by sex, with 8h-TRF delaying the median health report onset by 52 days in males compared to AL controls (Log-rank Mantel-Cox, P = 0.0018) (Fig. 4E). Additionally, while there were no significant differences under TRF, overall, females spent significantly fewer days on health report until death than males (two-way ANOVA: feeding, P > 0.05; sex, P < 0.01; interaction, P > 0.05) (Fig. 4F). Altogether this demonstrates there is sex-specificity in the development of and resistance to disease.

### 8h-TRF extends median and maximal lifespan in males

**Figure 5:**
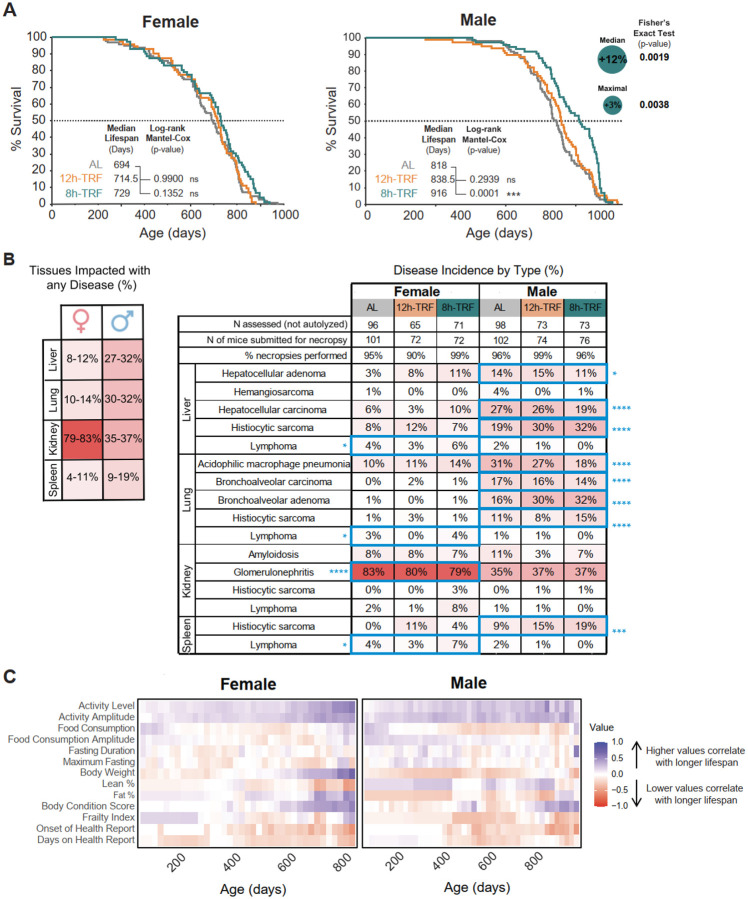
8h-TRF extends lifespan in males. (**A**) Kaplan-Meier survival curves and day median lifespan reached. Log-Rank Mantel-Cox Test for significant difference in overall survival TRF vs AL. Fisher’s exact test for median and maximal survival TRF vs AL. Females N=72–99 per group. Males N=73–99 per group. (**B**) (*Left*) Histopathology results showing the percentage incidence of any disease type in the most impacted tissue types between females and males and (*Right*) incidence by specific disease type within these tissues separated by sex and feeding group (Full table with all tissues and diseases shown in Tables S4–6). Significant differences females vs males highlighted by blue boxes and shown with blue asterisks (P < 0.05) as determined by contingency tables and Fisher’s exact tests. (**C**) Heatmaps of correlations between lifespan and 13 health measures recorded throughout the experiment. Colors range from blue (parameters where higher values correlate with longer lifespan) to red (parameters where lower values correlate with longer lifespan).

For the first time, we were able to investigate the impact circadian aligned TRF with regular chow diet, on lifespan in mice (Fig. 5A, and Tables S1–3). In females, 12h-TRF alone did not extend median lifespan, with females surviving to a median of 715 days compared to 694 days in AL. However, 8h-TRF with self-imposed CR resulted in a ~5% increase in median lifespan to 729 days (Fisher’s exact test, P = 0.0442), though overall survival curves showed no significant difference. In males, 12h-TRF also failed to extend lifespan, with a median survival of 839 days compared to 818 days in AL, a finding now reproduced across two independent experiments (Fig. S9). Interestingly, 8h-TRF with lifelong self-imposed CR, significantly improved overall survival for males (Log-rank Mantel-Cox, P = 0.001). Median lifespan increased by approximately 12%, reaching 916 days (Fisher’s exact test, P = 0.0019), and maximal lifespan was prolonged by about 3% (Fisher’s exact test, P = 0.0038). Though TRF is beneficial for improving health in both sexes, its capacity to extend lifespan appears to be feeding window- and sex-dependent.

### Sex-dependent differences in disease types and incidence at death

After death each mouse underwent gross necropsy and histopathological analysis by a veterinary pathologist to determine the types of diseases that contributed to its debilitation (Fig. 5B, and Tables S4–6). As shown previously (10), the types and relative frequency of diseases were similar for males across all feeding conditions with the most common cause of death or debilitation being neoplasia. Along with the delayed onset of health reports, this supports that rather than preventing disease, 8h-TRF delays the development of age-related diseases in males. TRF did not reduce the overall occurrence of disease in females. However, females developed neoplasms far less frequently than males (Fisher’s exact tests females vs males: Adenoma in liver, P < 0.05; Carcinoma in liver and lung, each P < 0.0001; Histiocytic sarcoma in liver and lung, each P < 0.0001; Histiocytic sarcoma in spleen, P < 0.001; Acidophilic macrophage pneumonia in lung, P < 0.0001; Adenoma in lung, P < 0.0001). Instead, most females were debilitated by glomerulonephritis in the kidneys and had a higher incidence of lymphoma than males (Fisher’s exact tests females vs males: Lymphoma in liver, kidney, spleen, each P < 0.05; Glomerulonephritis, P < 0.0001). These results have revealed pronounced differences in neoplasia and development of other age-related morbidities between the sexes.

### Sex-dependent strategies for achieving a healthy lifespan

Identifying biomarkers of health that correlate with lifespan is crucial both for understanding the aging process and for evaluating the effectiveness of interventions. Here, we correlated 13 health measures recorded throughout each mouse’s life, including measures of behavior, body physiology, frailty, and disease (Fig. 5C). In both sexes, higher activity levels and amplitudes consistently correlated with longer lifespan, especially at older ages. Although voluntary wheel running does not extend lifespan (44), these findings support activity as a valuable healthspan biomarker (10).

In females, lower food consumption and amplitude during midlife, but higher values in early life and late life correlated with longevity. Shorter fasting durations and lower maximum fasting times (i.e., longer feeding windows) also showed modest associations. While not formally quantified, we frequently observed a marked consolidation of feeding shortly before death, potentially explaining this trend. Conversely, males tended to correlate longer lifespan with higher feeding amplitude, longer fasting durations, and greater maximum fasting times during early to midlife, with this trend reversing in late life.

Body physiology measures also showed sex-specific correlations. In females, lower lean mass and body weight in early-life correlated with longer lifespans, but higher body weight, fat composition, and body condition scores correlated with longevity in late life. Males showed a modest trend for higher fat in late-life as well but reversed patterns for body weight, condition score, and lean mass. The late-life fat retention trend aligns with studies suggesting fat provides protective health benefits in old age (48). However, the opposing body mass patterns indicate sex-specific strategies for maintaining healthy physiology where males appear to benefit from limiting weight gain, while females need to maintain higher body weights in late life, to support longevity.

Frailty index showed inconsistent correlations with lifespan in females, whereas in males, lower frailty scores consistently associated with longer lifespan (49). For both sexes, delayed onset and shorter duration of veterinary health reports correlated with increased longevity, indicating that delayed and reduced disease burden supports extended lifespans.

### Healthspan is more prolonged in females and can be extended by 12h-TRF

**Figure 6:**
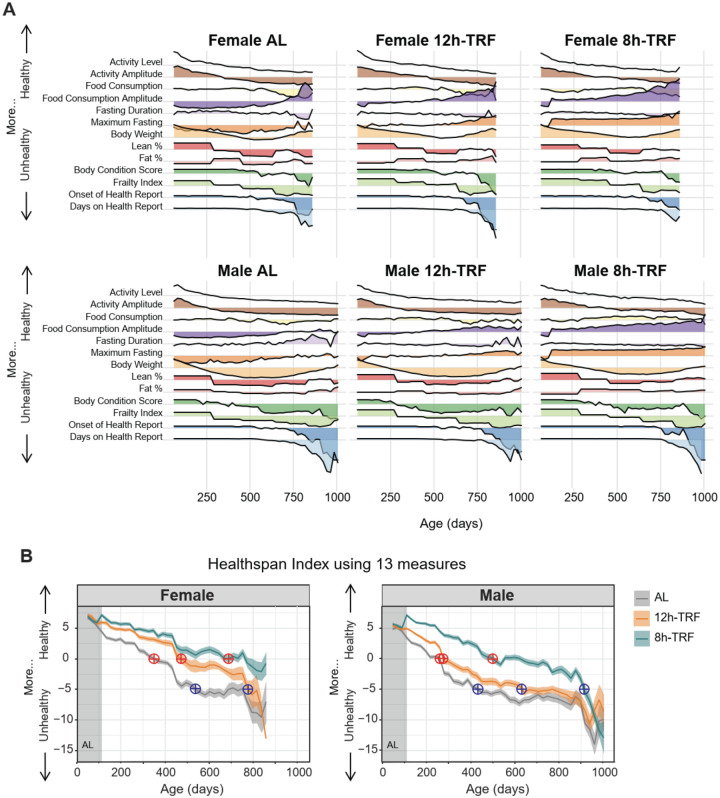
TRF confers greater healthspan benefits to females. (**A**) Ridgeline plots for each of the 13 health measures recorded throughout the experiment. All parameters were standardized (mean = 0, SD = 1) and, where appropriate, oriented in the positive direction to reflect better health. (**B**) Healthspan index summing the 13 health measures for each group from panel A. Higher values indicate better health and lower values poorer health. Red crosshairs mark the age at which each group’s index crosses 0; blue crosshairs mark when it crosses −5.

While TRF improved and attenuated age-related changes in several markers of health, we next aimed to quantify both the magnitude and duration of its overall impact by measuring healthspan. Frailty index is commonly used to determine healthspan (47, 49), but it cannot incorporate the impact of behavioral changes in wheel-activity and food consumption and can miss important physiological changes in the time between assessments. To generate a continuous healthspan index, we integrated frailty with 12 additional health measures (the same 13 in Fig. 5C) by first normalizing each data set (mean = 0, standard deviation = 1), then scaling the parameter directionally by providing context based on whether higher or lower values reflected better health (e.g., high wheel-running activity was scaled positively, while low frailty scores were inverted and also scaled positively) (Fig. 6A), and finally summing the values across age points (Fig. 6B). With higher values reflecting better health, females not only begin in a healthier state than males during the AL baseline but also cross the subjective thresholds of ‘0’ (red crosshairs) and ‘–5’ (blue crosshairs) 100–200 days later than their male counterparts in the same feeding group. Importantly, we find that in females at ‘0’, 12h-TRF is sufficient to extend healthspan by more than 100 days relative to AL, while in males both AL and 12h-TRF cross this threshold at similar ages. In both sexes 8h-TRF extends healthspan across this threshold by more than 200 days compared to AL. Together, these results suggest that although females do not live longer under TRF, they remain healthier for significantly longer.

## Discussion:

Previous studies have established that TRF improves circadian rhythms, ameliorates metabolic syndrome, and improves overall health in obesity models consuming a ‘Western diet’ high in fats and sugars (25–29). We expand on these findings by revealing that circadian aligned TRF confers significant, long-term health benefits even under normal nutritional conditions. We show that, depending on the feeding window and sex, TRF prolonged higher amplitudes of daily rhythms in feeding and wheel-running, which are known to feed back on the circadian clock to drive robust molecular and behavioral oscillations (14, 15, 17, 50–53). Although the mice were not obese, the intervention still significantly reduced age-related increases in weight and fat mass. TRF similarly slowed the age-related decline in lean composition, which may also contribute to the observed improvements in adiposity and suggests that TRF can delay the onset of functional disabilities (54–56). We also present novel findings that both TRF windows reduce frailty index in females and males, as in CR and intermittent fasting studies (57–61). Altogether, this demonstrates that TRF does not merely act as a remedial strategy to ‘rescue’ poor health but can actively improve behavioral and physiological markers of health under normal metabolic aging conditions.

Our findings further suggest that, when the health measures recorded throughout the experiment are integrated into a comprehensive healthspan index, females are overall healthier than males. Moreover, timing alone through 12h-TRF is sufficient to extend healthspan in females, but only weakly so in males. Although 8h-TRF more noticeably improved healthspan in males, the benefits remained substantially greater in females. However, this approach may oversimplify sex-specific differences, as males and females exhibit distinct patterns of health deficits that correlate differently with longevity. Consequently, a single healthspan scale may not adequately reflect these complex, sex-dependent impacts on aging. Additionally, although females generally show better health scores, we found they had shorter lifespans than males, highlighting a potential disconnect between this healthspan index and ultimate survival outcomes. Regardless, this sex-specific response in healthspan likely reflects a combination of biological factors. Estrogen is known to confer broad protective effects on health and aging (62), which may explain the overall female advantage even under AL feeding, and is known to feedback onto the circadian clock (63–65). With recent literature showing that females possess a more robust circadian system (66), this may underlie their enhanced responsiveness to circadian-based interventions. Indeed, studies have found that females express a greater number of rhythmic genes, display higher amplitude rhythms, and are more sensitive to external cues (67–69). As such, TRF may more effectively enhance rhythms at the molecular level to promote health in females (18, 19).

For the first time, we show that aligning feeding to the active phase under 12h-TRF does not extend lifespan. However, a narrower feeding window of 8h-TRF along with self-induced CR conferred marginal or strong lifespan extension in females and males, respectively. These results contrast with previous studies showing that lifelong 20% CR, adjusted relative to age-related changes in AL feeding, extends lifespan by 40.6% in female and 24.4% in male C57BL/6J mice (70). While our 8h-TRF mice maintained ~20% CR for nearly half of their lifespan, both males and females gradually lessened the degree of CR relative to AL feeding shortly before reaching median lifespan. Despite this, 8h-TRF males gained a 12% extension in median lifespan, indicating that even a lower degree of CR is still impactful in males. In contrast, females appear to require a sustained degree of CR ≥20% to see lifespan benefits. Alternatively, the duration of fasting may be more crucial for female lifespan, as longer fasting periods with CR have enhanced lifespan effects in females (8, 70), but not in males (10). The mechanisms underlying these sex-dependent differences in response to CR and TRF are not fully understood, especially between different strains of mice (5, 70), but are likely influenced by a complex interplay of factors such as differences in nutrient sensing and metabolic pathways, DNA repair, and the effects of sex hormones (36, 66, 71).

Survival curves for AL mice unexpectedly showed an 18% increase in median survival for males compared to females, contrary to the common trend in mammalian studies where females typically live longer due to the genetic advantage of a backup X chromosome and the protective effects of estrogen (72, 73). However, in C57BL/6J mice, lifespan outcomes are inconsistent possibly due to genetic drift within the isogenic line as well as environmental variations between experiments (73–76). While genetic factors may play a role, the shorter lifespan in females could stem from enhanced cold sensitivity and higher thermoregulatory energy demands (77–80). The smaller bodied females, needing more energy to maintain body temperature as evidenced by the pronounced increase in food intake with age, may have been adversely affected by the harsher individual housing conditions without nesting material. Although lower body temperatures can extend lifespan (81, 82), cold stress can also divert energy away from and impair metabolic, immune, and reproductive functions (83, 84).

Health report monitoring and post-death histopathological analyses revealed significant differences between the sexes in the progression of age-related diseases, particularly in neoplasia. With the exception of lymphoma, female mice had significantly lower occurrences of neoplasms in the liver, lung, kidney, and spleen which supports a growing body of data that females have greater resistance to cancer (85–87). This resistance is largely attributed to sex differences in immunity, mutational burden, and DNA repair, as well as the protective role of X-linked tumor suppressor genes and their interaction with the p53 pathway in females. Females in this cohort were instead most susceptible to kidney failure due to glomerulonephritis, similar to a previous study (88). This condition has been linked to menopause in women, with its progression often accelerating after the decline in estrogen levels (89). In mice, this disease can be further exacerbated in the absence of the *estrogen receptor alpha* gene (90), emphasizing the protective role of estrogen signaling and uncovering renal disease as a potential biomarker of reproductive aging.

One limitation of this study was the inability of the automated feeders to retract or block a food pellet that goes uneaten beyond the restricted food intake window. This meant that often, a single pellet would be by the mouse, outside of its 12h or 8h time restricted feeding window. Additionally, some mice had the tendency to hoard pellets within their cage, leading again to food availability outside of the TRF window, a trait that has already been shown in aged mouse populations (39). However, a single daytime pellet accounted for ≤10% of total caloric intake, and after subtracting hoarded food, mice consumed ≥90% of the pellets taken from the feeder during the correct feeding windows. These findings, along with evidence that TRF on 5 out of 7 days per week regimens still improves metabolic health (34), suggest that moderate TRE adherence may still prolong healthspan and be compatible with modern human lifestyles. Additionally, since our TRF regimens were started in early adulthood and maintained throughout life, it is unclear whether late-life implementation would yield similar benefits. Short-term studies indicate that TRF in the elderly can reduce high-fat diet-induced inflammation and metabolic dysfunction, and studies with CR in aged mice still provided health and lifespan benefits (91–93). However, these benefits are not as strong as when started in early adulthood.

In conclusion, our study provides compelling evidence that early onset of 12h or 8h of circadian-aligned TRF can significantly improve healthspan, more potently so in females, and that 8h-TRF can extend lifespan in males. While this still warrants further exploration in additional mouse strains and at varying ages, altogether this highlights the potential of TRF as a practical, non-pharmacological intervention to prolong the health quality of human lifespans.

## Materials and Methods:

### Animals and Housing Conditions

264 male and 264 female C57BL/6J mice 6 weeks of age were ordered from Jackson Laboratories, Bar Harbor, ME. As done previously, starting at 2 months of age the mice were: **(i)** individually housed in standard polycarbonate cages with stainless steel running wheels inside isolation cabinets under light:dark (LD) of 12:12 hours, **(ii)** fed 300mg pellets of purified diet (F0075, Bio-Serv) using automated feeders with water provided *ad libitum* (AL), and **(iii)** cage changed every 21 days (9, 10, 94). The Institutional Animal Care and Use Committee (IACUC) of the University of Texas Southwestern Medical Center approved the animal protocol (APN 2015-100925), which has been renewed every 3 years (in 2018, 2021, and 2024).

### Time-Restricted Feeding (TRF) Regimens

At 2 months of age, mice were placed in individual cages as described above and fed *ad libitum* (AL) for 8 weeks using the automated feeder system (9, 10). At 4 months of age, the mice were divided into three feeding groups for the remainder of the experiment: **(i)** 108 of each sex continued in AL as a control group, **(ii)** 78 of each sex in a 12-hour TRF group where the automated feeder restricted food dispensing to the 12-hour night (12h-TRF, ZT12-24), and **(iii)** 78 of each sex in an 8-hour TRF group with food dispensing restricted to the middle 8 hours of the night (8h-TRF, ZT14-22). In addition to the timing of the feeding window, the feeders were also programmed with a 10 min delay between pellet dispensing to reduce hoarding behavior. Regardless of the feeding group, all mice had access to 22 pellets/day so as not to impose caloric restriction.

For a minimum detection of 10% life extension with 80% power a minimum of 96 animals were needed in the AL group and 72 in each of the TRF groups (95). Extra mice were included in each group to account for any that may not adapt to the feeder/diet or for non-aging-related deaths.

### Daily Monitoring of Feeding and Wheel-Running Activity

As before, the feeders were set and continuously recorded quantity and timing of food intake via ClockLab Chamber Control Software v3.401 (Actimetrics Inc., Wilmette, IL, USA) (9, 10). Uneaten food was removed from the bedding every 21 days during cage change, or weekly for chronic hoarders under TRF. The feeding data presented in Figure 1B and Supplemental figures S2 and S3 represent food consumption with hoarding removed. All other feeding figures represent the quantity and pattern of pellet taking from the feeder, not excluding hoarded food. Wheel-running activity was also continuously recorded as before using an updated version of ClockLab Data Acquisition System v3.604 (Actimetrics Inc., Wilmette, IL, USA).

### Body Weight and Composition Measurements

The body weight of each mouse was measured every 21 days during cage change (morning, ZT4-7) throughout its lifespan. Body composition measurements of lean and fat mass (g) were assessed every 6 months during cage change (ZT4-7) using a 100H-EchoMRI Body Composition Analyzer (EchoMRI, Houston, TX, USA).

### Frailty Scoring

Every 6 months the mice were assessed for frailty, or physical vulnerabilities, according to 31 parameters of aging modified from Whitehead et al. (47). These parameters include a range of physiological evaluations, scoring coat condition, ocular and auditory responses, physical and musculoskeletal state, respiration rate, body weight, and temperature. Each assessment was scored either 0=absent, 0.5=mild, or 1=severe. The scores were totaled to calculate an overall index score.

### Health Monitoring and Survival Study

Mice were physically health checked every 21 days during cage change and visually inspected every 10 days during refilling of the feeders. Using the feeding and wheel monitoring software, mice were also checked virtually every day and only physically health checked if feeding fell <5 pellets and/or wheel running fell below set thresholds (1.14 counts/min within 24hr, reduced by 10% every 6 months as the mice age) as performed previously (10).

If any mouse was found to have a mild/moderate health ailment (e.g. dermatitis, external tumor, abdominal mass, head tilt) or a body condition score < 3 (Scored 1–5 with: 1 being emaciated, 3 ideal condition, and 5 obese) it was placed on health report. These records were also used to generate Figures 4D–F. Mice on health report were physically health checked weekly by the UT Southwestern Animal Resource Facility (ARC) veterinary staff and twice a week by our own laboratory staff. In order to obtain accurate but humane lifespan data, mice were monitored carefully for euthanasia criteria that would indicate imminent death (moribund) or unacceptable levels of pain (analgesics were avoided to avoid confounding the results). Criteria were determined in conjunction with the ARC veterinarians according to AAALAC guidelines and include: Non-responsive to touch, hypothermic or cold to touch, slow or labored breathing, failure or inability to eat/drink after moist chow is given, a body condition score of 1, body weight loss of >20% from baseline or between cage changes, a broken/fractured/dislocated limb, head tilt that is resulting in an animal being unable to maintain sternal body position which would affect their ability to consume food and water, severe eye protrusion/infection, severe dermatitis, grade 3 rectal or penile prolapse, urinary obstruction, or a subcutaneous tumor >2cm.

The number and date of those euthanized or found dead across the feeding groups and sexes were recorded and used to generate the Kaplan-Meier survival curves. A total of 35 animals (35/528) were censored from the survival curve if death occurred earlier than 6 months or due to non-aging related injuries or death: 9 AL females, 6 12h-night TRF females, 6 8h-night TRF females, 9 AL males and 5 8h-night TRF males.

### Necropsy and Histopathology

Mice euthanized or found dead were submitted for gross necropsy and blinded histopathological analysis was performed, as done previously (10). Less than 10% of the animals submitted were too autolyzed to analyze. Disease types were tallied together by tissue type, sex, and feeding group.

### Health Measures vs Lifespan Correlation

Throughout the experiment we recorded thirteen health measures including: wheel-running activity to determine overall activity level and diurnal amplitude, food consumption to determine overall level, diurnal amplitude, average fasting duration, and maximum fasting times, body weight, composition of lean and fat mass as a percentage of body weight, body condition score, frailty index, and the onset and duration of veterinary health reports.

### Healthspan Index

Healthspan index was calculated by summing the calculated scaled values of each of the thirteen health measures. In the case of body condition score, an absolute difference from BCS=3 was calculated. For body fat percentage, an absolute difference in value from 24.45% was calculated. The value 24.45% was used based on previous literature showing the average body fat percentage across multiple healthy mouse strains at the age of 16 weeks (96). All measures were then normalized to a mean of 0 and a standard deviation of 1, within each measure. Certain values were then inverted, given their context, in order to have positive numbers representing a healthier state, and negative values representing an unhealthy state. For example, days_on_hr is inverted, as higher values would be less healthy, and lower values are healthier. All normalized values were then summed together at 21-day intervals.

### Statistical Analyses

As performed previously, all feeding and wheel-running activity was collected using Chamber Control and ClockLab, respectively (Actimetrics, Inc) (10). Each point on the longitudinal profiles represents a 21-day mean. Twenty-four-hour profiles were binned by hour, with each age point reflecting a 15-day mean (taken from the middle of each 21-day cage cycle to avoid behavioral changes due to cage change stress). For each mouse, each 15-day bin is analyzed by 1-dimensional Discrete Fast Fourier Transform (FFT) using the mixed-radix algorithm for real-valued input (Python function from the scipy library: scipy.fft.rfft) (97). The Blackman window function is applied to the data prior to FFT analysis to inhibit spectral leakage (98). The power of each frequency component is then normalized, with the total normalized power of all frequency components equaling 100. The normalized power from the 24h frequency component is then taken as the diurnal amplitude of either food intake behavior or running wheel activity. This value represents the percentage of contribution of the 24h frequency component, to the entire 15-day behavioral period. For example, if the diurnal amplitude for food intake is 21, it indicates that 21% of the observed variation in food intake can be directly attributed to the animal’s consistent 24-hour daily cycle. The output of this analysis for each mouse was then averaged within group, to provide a group-wise indication of diurnal amplitude.

Cumulative health report and survival curves were analyzed using Log-rank Mantel Cox and Fisher’s exact test, health report type and necropsy report disease type percentages analyzed by contingency tables and Fisher’s exact test and, and all other plots analyzed using two-way ANOVA with Tukey’s or Holm’s post-hoc analyses (10, 99). Data plots were generated and statistically analyzed by Prism 10 (Graphpad Software Inc), Python, and R.

## Supplementary Material

Supplement 1

Supplement 2

1

Materials and Methods

Figures S1 to S9

Tables S1 to S6

## Data Availability

Data are available in the main text or the supplementary materials
