# Supplementary material for: Time-Restricted Feeding Extends Healthspan in Female and Lifespan in Male C57BL/6J Mice
Source: bioRxiv. 2025 Oct 23:2025.10.22.683527. Preprint. [Version 1] doi: 10.1101/2025.10.22.683527 (PMC12633347; doi:10.1101/2025.10.22.683527)
Supplement: 1 [file NIHPP2025.10.22.683527V1-supplement-1.pdf]

bioRxiv preprint doi: https://doi.org/10.1101/2025.10.22.683527; this version posted October 23, 2025. The copyright holder for this preprint (which was not certified by peer review) is the author/funder, who has granted bioRxiv a license to display the preprint in perpetuity. It is made available under aCC-BY 4.0 International license.

Supplemental Figures and Legends:

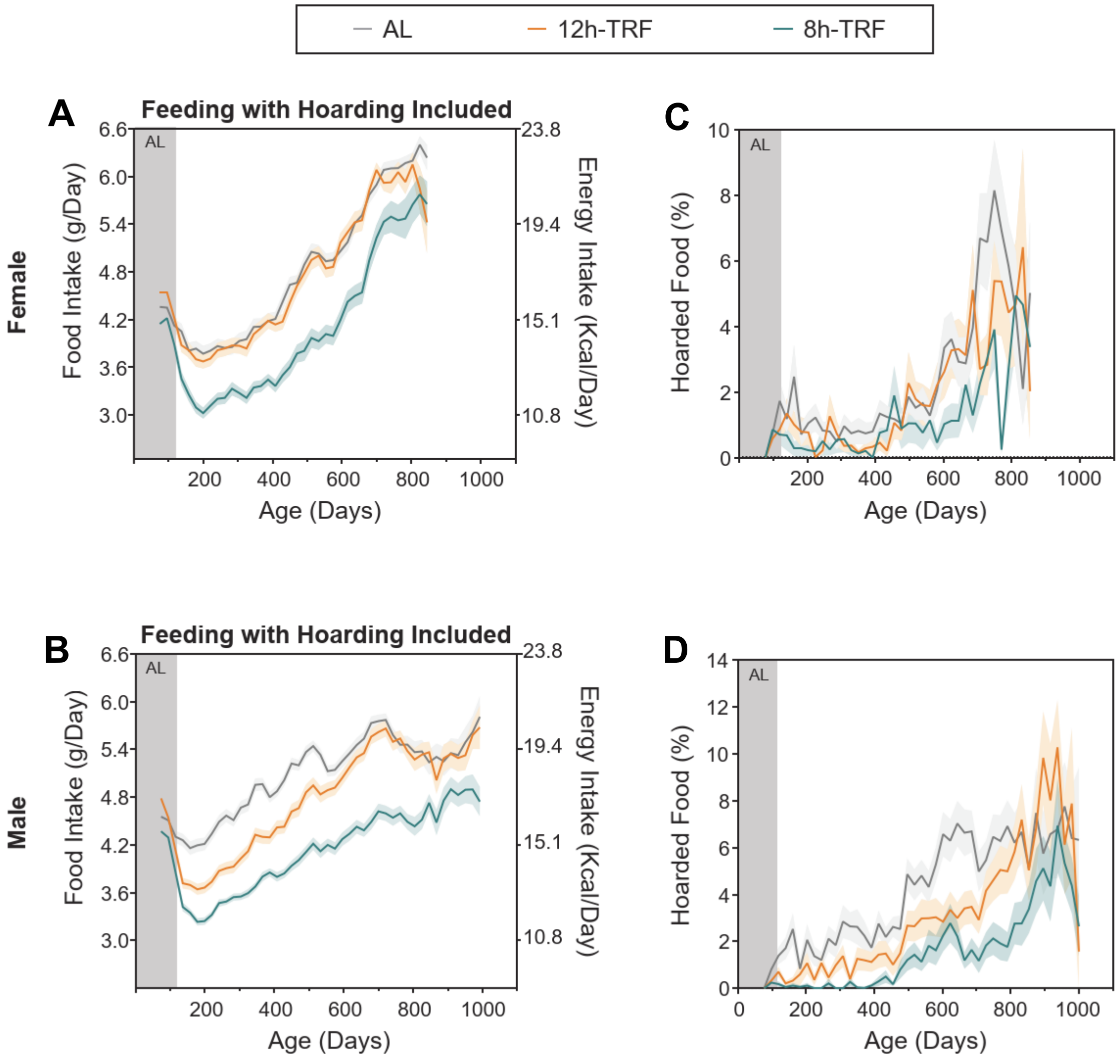

Figure S1: Food taking and hoarding behavior: (A) In females and (B) males, longitudinal profiles of daily food taken from the feeder including uneaten (hoarded) food found in the cage (g/Day) and the equivalent energy intake (Kcal/Day). (C) In females and (D) males, longitudinal profiles of the percent (%) of hoarded food in the cage. Mean of 21 days ± SEM (shaded regions). *Ad libitum* (AL) baseline feeding (Gray area). Time-restricted feeding (TRF) (White area).

bioRxiv preprint doi: https://doi.org/10.1101/2025.10.22.683527; this version posted October 23, 2025. The copyright holder for this preprint (which was not certified by peer review) is the author/funder, who has granted bioRxiv a license to display the preprint in perpetuity. It is made available under aCC-BY 4.0 International license.

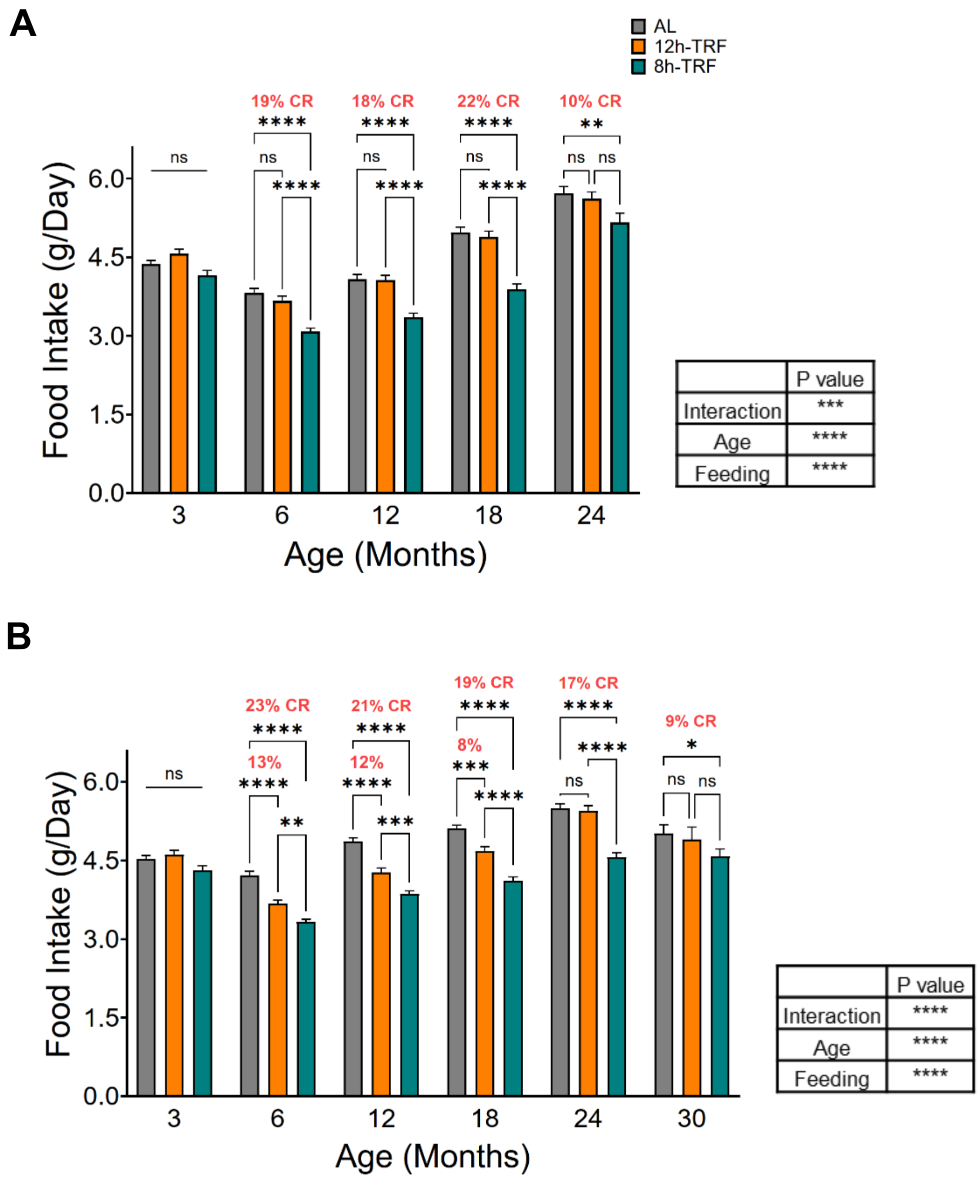

Figure S2: Food intake by monthly age epochs: (A) In females and (B) males, comparison of food intake with hoarding removed (g/Day) at 3 months of age and then every 6 months. Mean of 21 days at each age point ± SEM. Any caloric restriction (CR) relative to the *Ad Lib* control at each age epoch shown as %. Two-way ANOVA, Tukey's post-hoc.

bioRxiv preprint doi: https://doi.org/10.1101/2025.10.22.683527; this version posted October 23, 2025. The copyright holder for this preprint (which was not certified by peer review) is the author/funder, who has granted bioRxiv a license to display the preprint in perpetuity. It is made available under aCC-BY 4.0 International license.

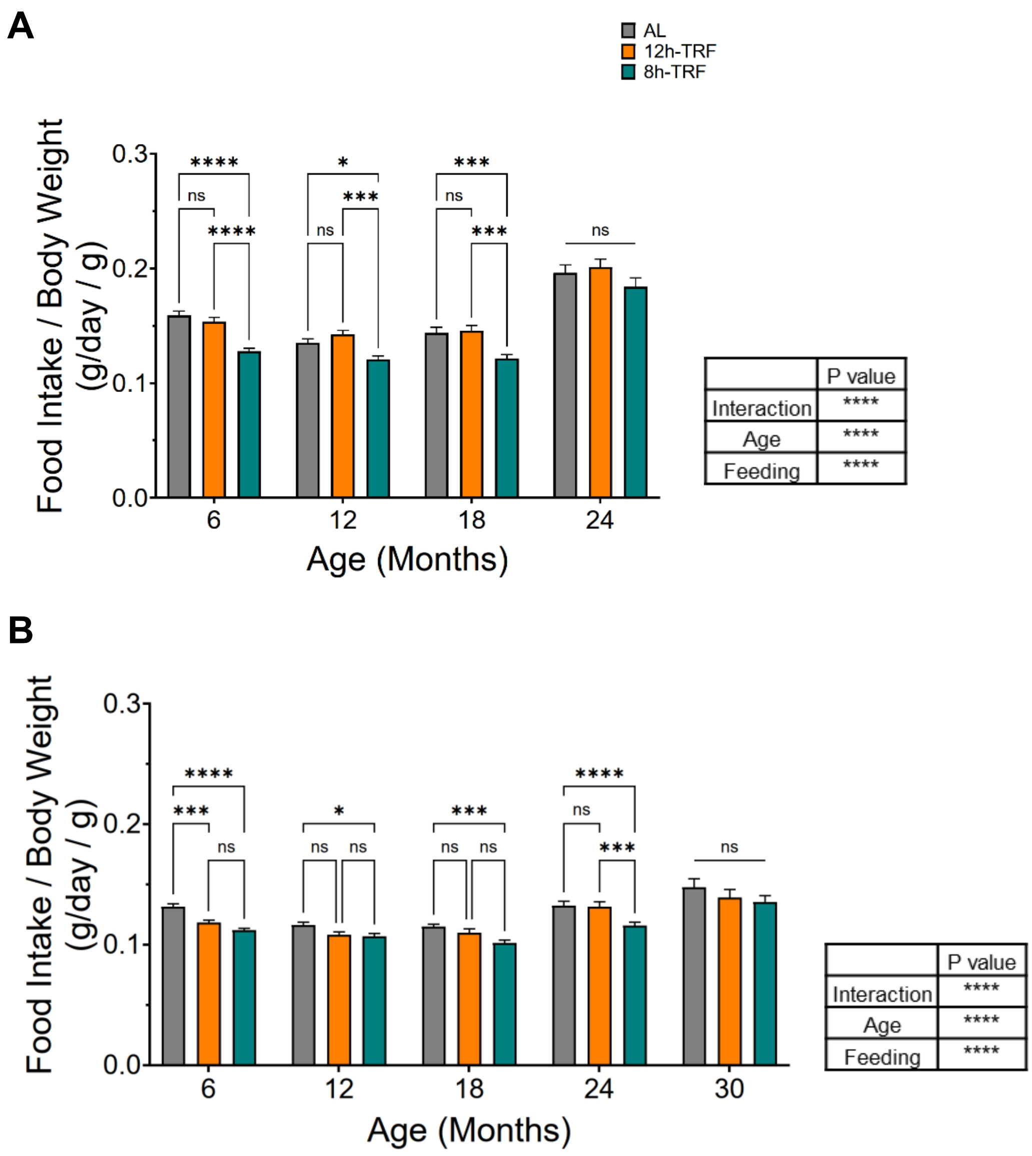

791 **Figure S3: Food intake relative to body weight by monthly age epochs:** (A) In females and (B)
792 males, comparison of mean food intake (g/day) with hoarding removed relative to body weight (g)
793 ± SEM at 3 months of age and then every 6 months. Two-way ANOVA, Tukey's post-hoc.

bioRxiv preprint doi: https://doi.org/10.1101/2025.10.22.683527; this version posted October 23, 2025. The copyright holder for this preprint (which was not certified by peer review) is the author/funder, who has granted bioRxiv a license to display the preprint in perpetuity. It is made available under aCC-BY 4.0 International license.

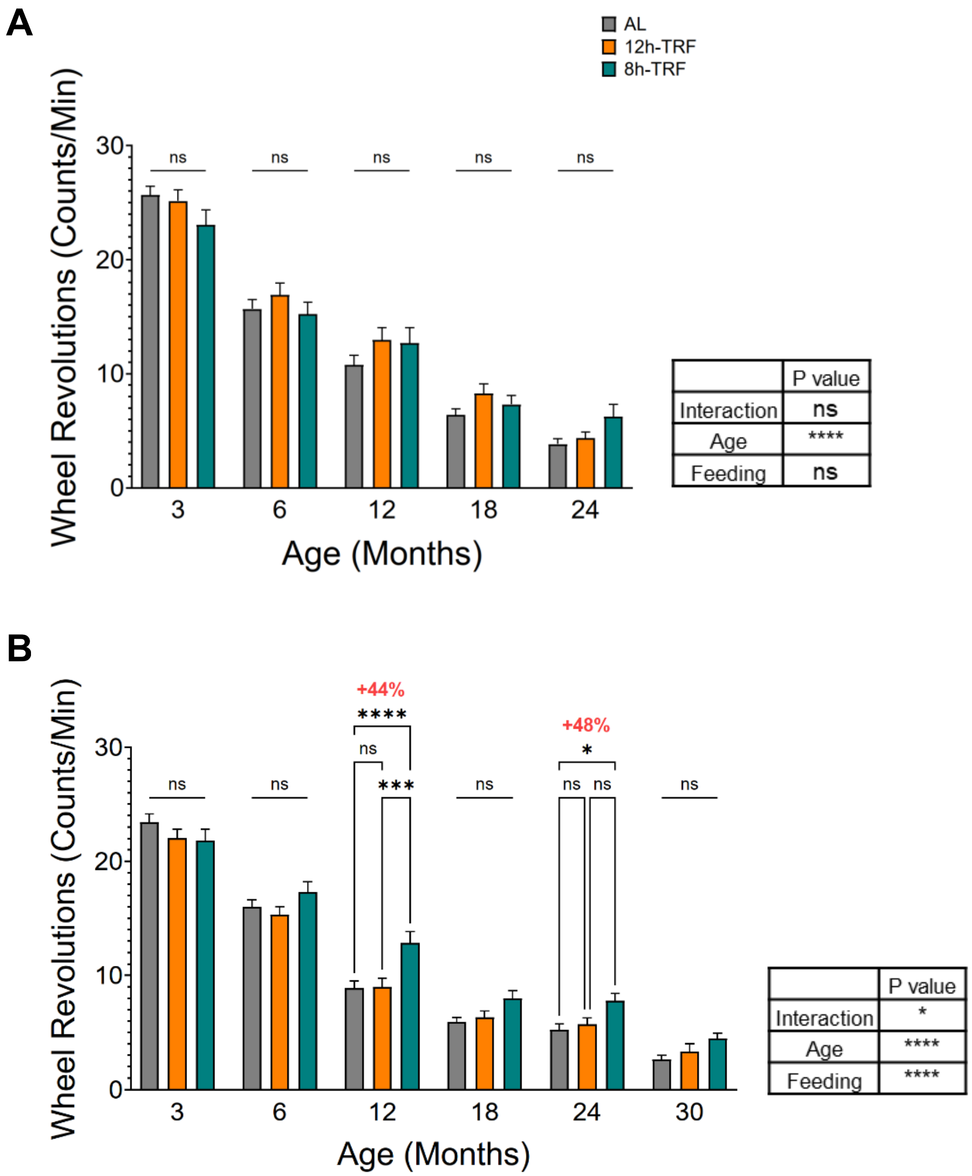

794 **Figure S4: Wheel activity by monthly age epochs:** (A) In females and (B) males, comparison of
795 daily wheel revolutions (Counts/Min) at 3 months of age and then every 6 months. Mean of 21
796 days at each age point ± SEM. Two-way ANOVA, Tukey's post-hoc.

bioRxiv preprint doi: https://doi.org/10.1101/2025.10.22.683527; this version posted October 23, 2025. The copyright holder for this preprint (which was not certified by peer review) is the author/funder, who has granted bioRxiv a license to display the preprint in perpetuity. It is made available under aCC-BY 4.0 International license.

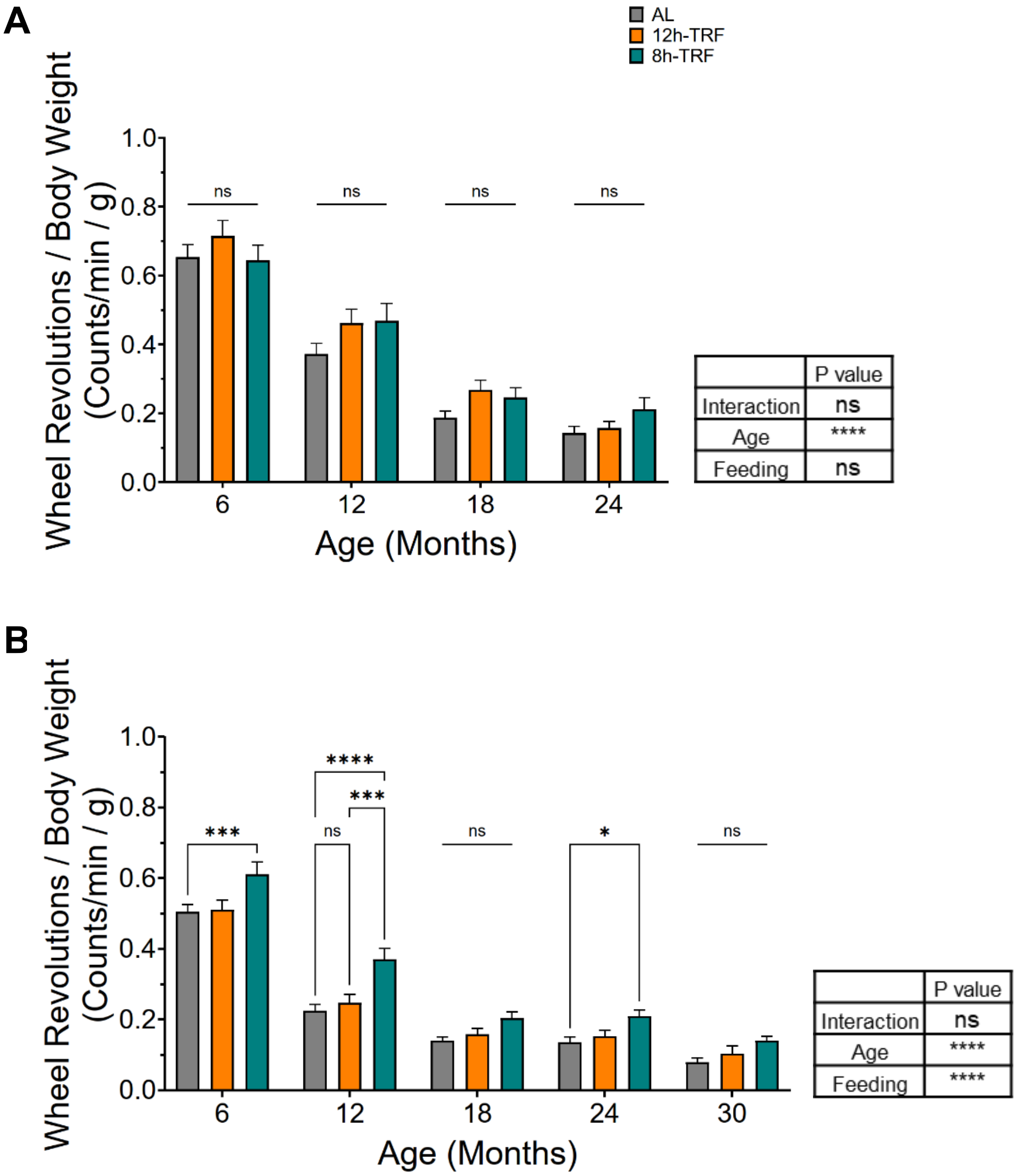

797 **Figure S5: Wheel activity relative to body weight by monthly age epochs:** (A) In females and
798 (B) males, comparison of mean wheel revolutions (counts/min) relative to body weight (g) ± SEM
799 at 3 months of age and then every 6 months. Two-way ANOVA, Tukey's post-hoc.

bioRxiv preprint doi: https://doi.org/10.1101/2025.10.22.683527; this version posted October 23, 2025. The copyright holder for this preprint (which was not certified by peer review) is the author/funder, who has granted bioRxiv a license to display the preprint in perpetuity. It is made available under aCC-BY 4.0 International license.

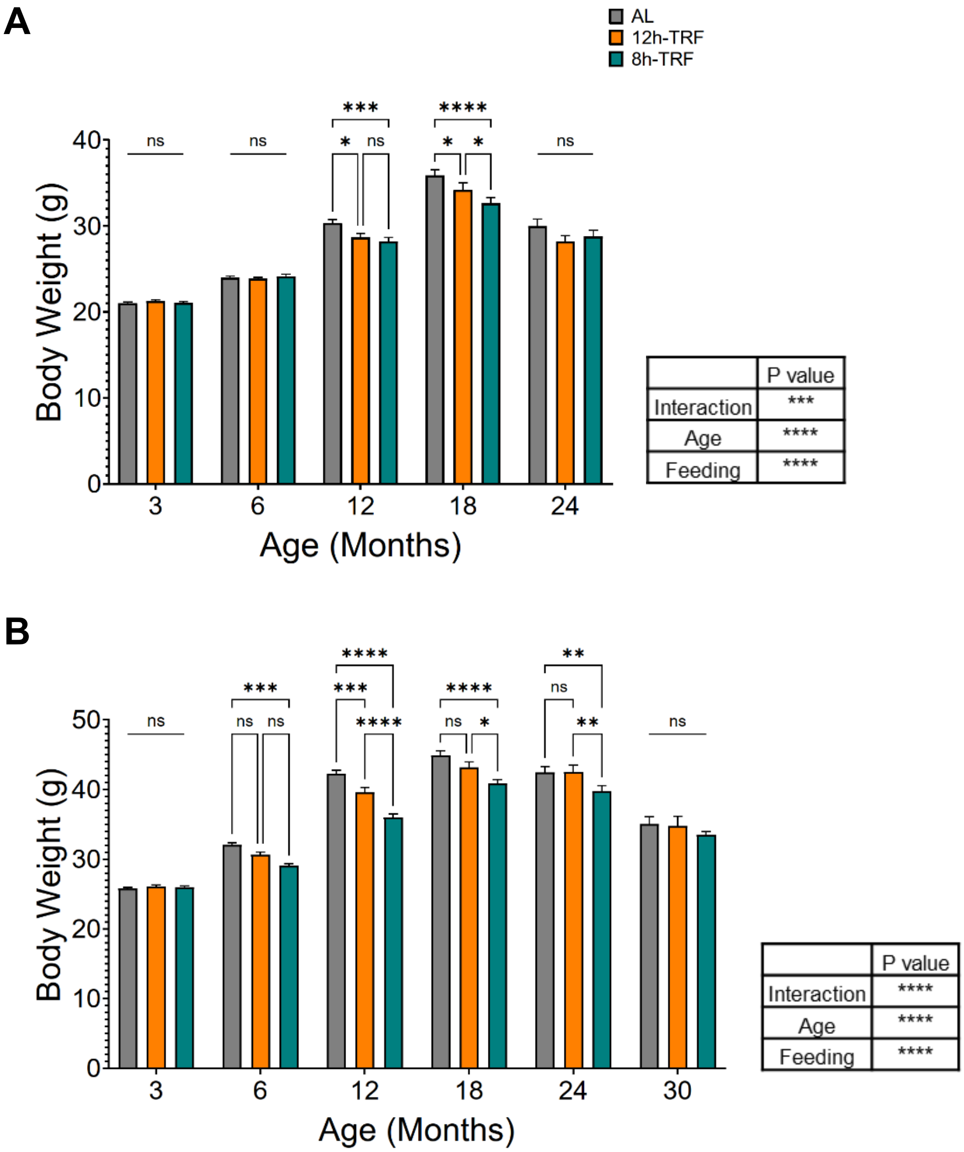

Figure S6: Body weight by monthly age epochs: (A) In females and (B) males, comparison of mean body weight (g) ± SEM at 3 months of age and then every 6 months. Two-way ANOVA, Tukey's post-hoc.

bioRxiv preprint doi: https://doi.org/10.1101/2025.10.22.683527; this version posted October 23, 2025. The copyright holder for this preprint (which was not certified by peer review) is the author/funder, who has granted bioRxiv a license to display the preprint in perpetuity. It is made available under aCC-BY 4.0 International license.

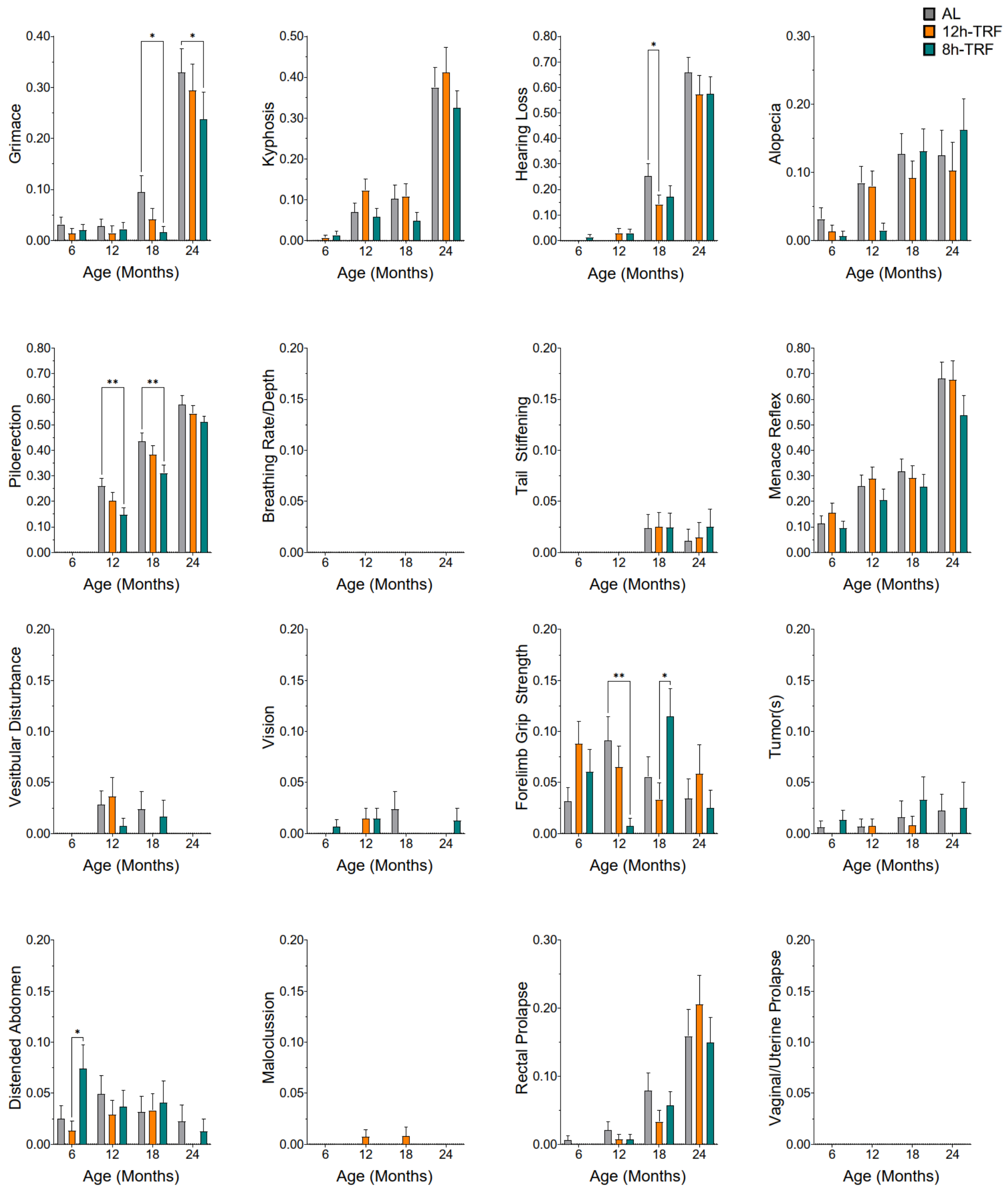

803 **Figure S7: Individual frailty parameter scores in females.** Mean frailty scores ± SEM every 6
804 months of age in females for each of the 31 parameters modified from (*47*). AL, N=44-79. 12h-
805 TRF, N=34-74. 8h-TRF, N=40-74. Two-way ANOVA, Tukey's post-hoc.

bioRxiv preprint doi: https://doi.org/10.1101/2025.10.22.683527; this version posted October 23, 2025. The copyright holder for this preprint (which was not certified by peer review) is the author/funder, who has granted bioRxiv a license to display the preprint in perpetuity. It is made available under aCC-BY 4.0 International license.

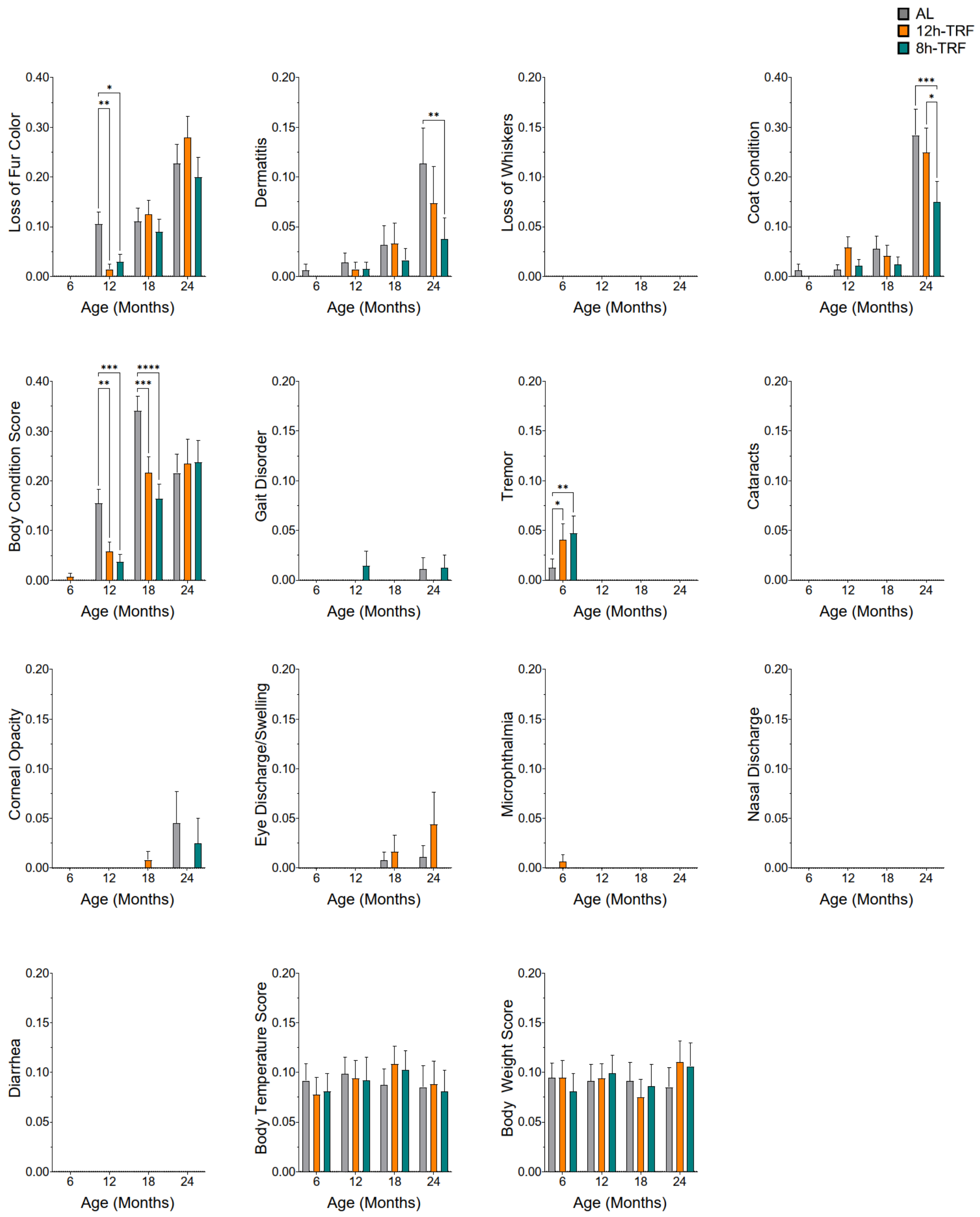

806 **Figure S7: Individual frailty parameter scores in females (Continued).**

bioRxiv preprint doi: https://doi.org/10.1101/2025.10.22.683527; this version posted October 23, 2025. The copyright holder for this preprint (which was not certified by peer review) is the author/funder, who has granted bioRxiv a license to display the preprint in perpetuity. It is made available under aCC-BY 4.0 International license.

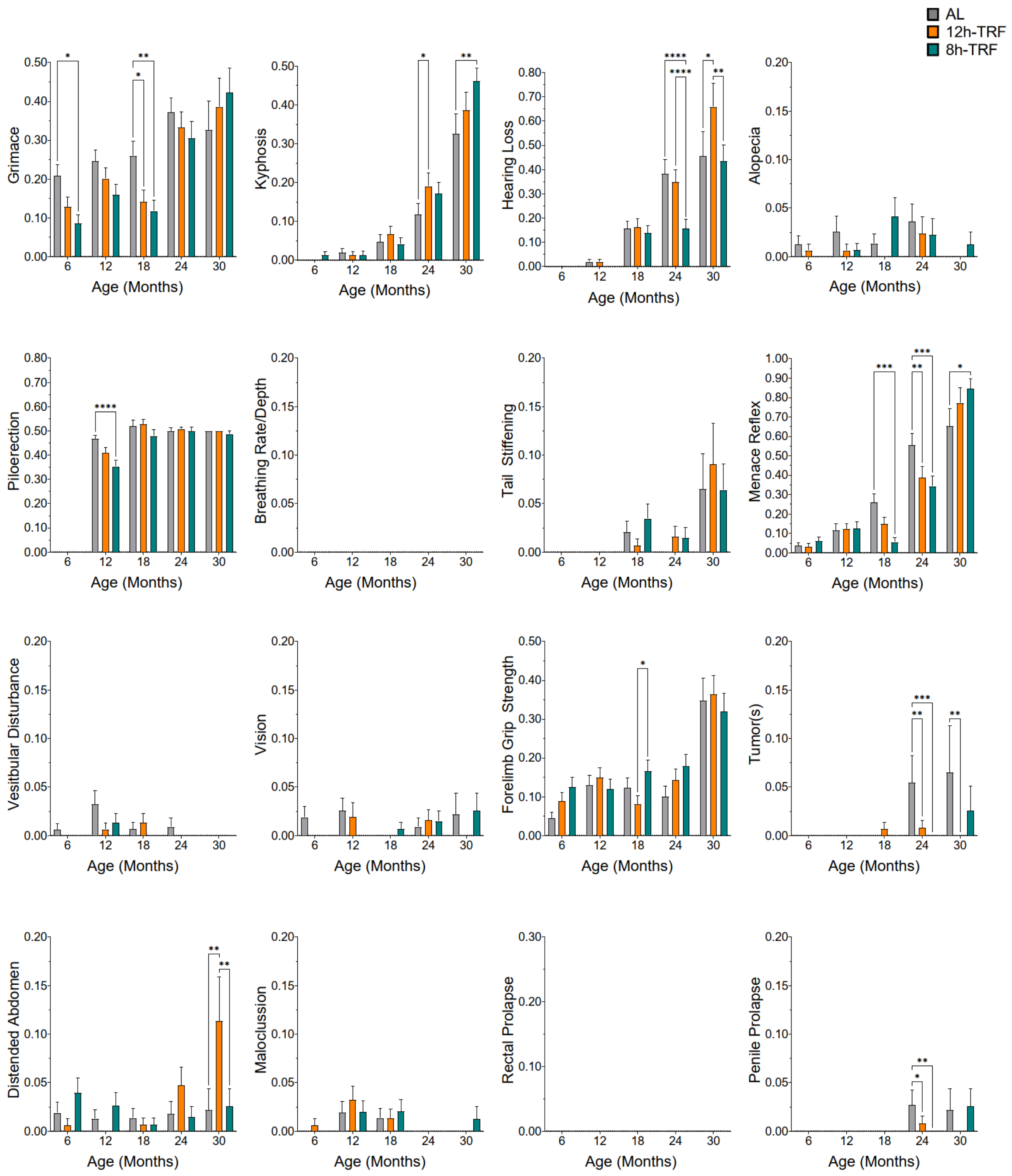

Figure S8: Individual frailty parameter scores in males. Mean frailty scores ± SEM every 6 months of age in males for each of the 31 parameters modified from (*47*). AL, N=23-79. 12h-TRF, N=22-78. 8h-TRF, N=39-76 Two-way ANOVA, Tukey's post-hoc.

bioRxiv preprint doi: https://doi.org/10.1101/2025.10.22.683527; this version posted October 23, 2025. The copyright holder for this preprint (which was not certified by peer review) is the author/funder, who has granted bioRxiv a license to display the preprint in perpetuity. It is made available under aCC-BY 4.0 International license.

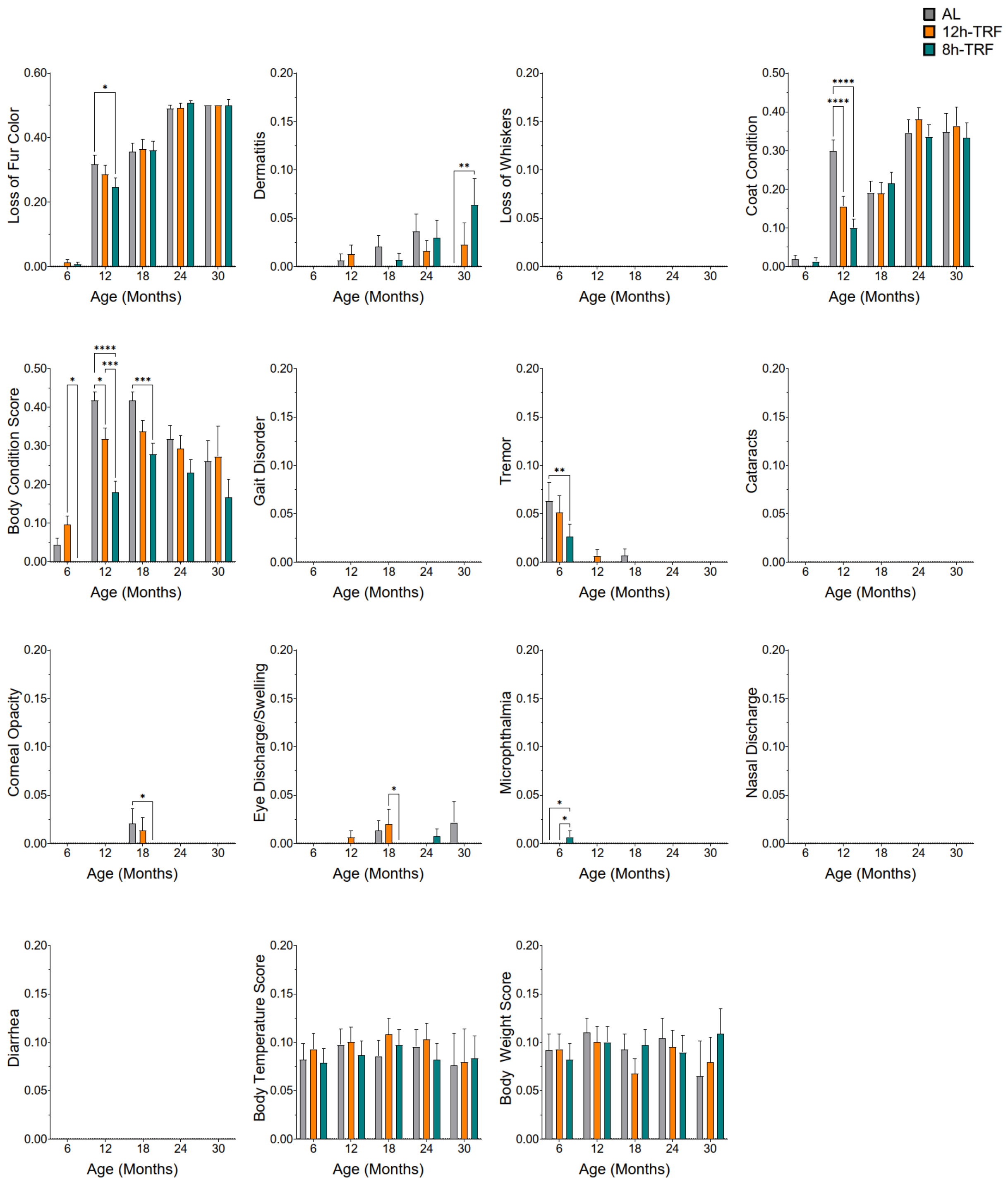

810 **Figure S8: Individual frailty parameter scores in males (Continued).**

bioRxiv preprint doi: https://doi.org/10.1101/2025.10.22.683527; this version posted October 23, 2025. The copyright holder for this preprint (which was not certified by peer review) is the author/funder, who has granted bioRxiv a license to display the preprint in perpetuity. It is made available under aCC-BY 4.0 International license.

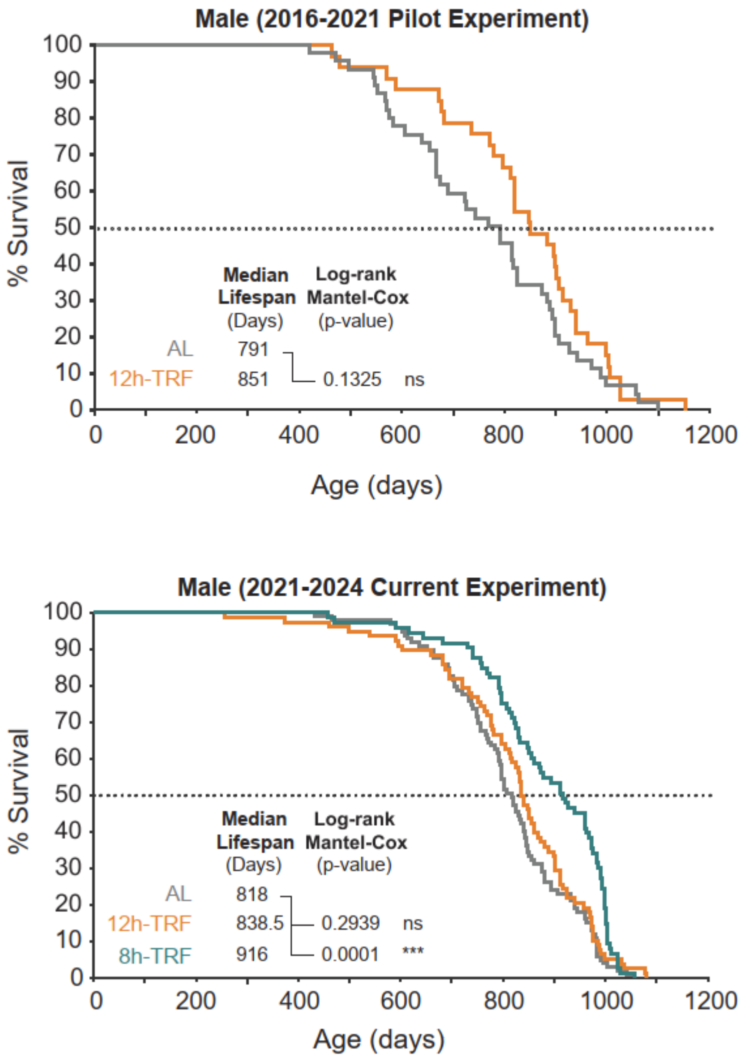

Figure S9: Repeat experiments show 12h-TRF does not extend lifespan in males: Kaplan-Meier survival curves and day median lifespan reached in a pilot experiment vs our current experiment. Log-Rank Mantel-Cox Test for significant difference in overall survival TRF vs AL. Fisher's exact test for median and maximal survival TRF vs AL. Pilot experiment: AL, N=43. 12h-TRF, N=33. Current experiment: AL, N=99. 12h-TRF, N=78. 8h-TRF, N=73.
